# Effects of sodium fluoride on serum concentrations of selected psychotropic drugs

**DOI:** 10.1111/1556-4029.70081

**Published:** 2025-05-25

**Authors:** Jennifer Liut, Julian Prangenberg, Michael Krämer, Alexandra Maas‐Gramlich, Peter Heese, Markus Banger, Burkard Madea

**Affiliations:** ^1^ Institute of Forensic Medicine, Forensic Toxicology University Hospital Bonn Bonn Germany; ^2^ Department of Addiction Disorders and Psychotherapy LVR Hospital Bonn Bonn Germany

**Keywords:** blood, fluoride‐stabilized, forensic toxicology, hemolysis, preservative, serum, sodium fluoride

## Abstract

Caution is imperative when interpreting drug concentrations in blood, plasma, or serum, given the potential variance in the distribution of a compound across these fluids. Preservatives, such as sodium fluoride, prove beneficial in preventing drug degradation in collected blood samples. This study aimed to examine the impact of sodium fluoride on different psychotropic drugs in both serum stabilized with and without this preservative. Paired blood samples (*n* = 100) were collected at the same time (with and without fluoride) from patients undergoing psychiatric treatment. Samples were examined for different compounds including antidepressants, antipsychotics, antiepileptics, and benzodiazepines using routine liquid chromatography–tandem mass spectrometry methods. Results were statistically evaluated and tested by using a paired *t*‐test (*α* = 0.05) in order to evaluate possible differences between drug concentrations in samples obtained from blood with or without fluoride. Median concentration ratios (fluoride‐stabilized/fluoride‐free) of all examined drugs ranged from 0.70 to 1.13 in the patient samples. For most substances exhibiting concentration ratios less than 1, the results indicated that concentrations in the samples with fluoride were, on average, 6%–30% lower than in samples without fluoride. Differences between drug concentrations in the patient samples were mainly attributed to matrix effects and fluoride‐induced hemolysis. The latter causing a shift in erythrocytes resulting in serum/plasma dilution, leading to under−/overestimation of a drug. The findings of this study can provide guidance for the interpretation of drug concentrations for therapeutic drug monitoring or assessment of acute drug‐induced impairment in cases were fluoridated serum/plasma is used.

**Highlights**

- The effect of sodium fluoride on serum drug concentrations is described.
- Median concentration ratios of the examined compounds are mostly lower in fluoride‐stabilized serum.
- When using fluoride‐stabilized samples, the blood/plasma ratio should be considered interpreting drug results.
- Toxicological evaluation should consider possible effects of preservatives on drug concentrations.

## 1 INTRODUCTION

Blood is often the biological matrix of choice for answering forensic and clinical questions about the acute effects of substances. Determining blood, plasma, or serum (both obtained by centrifugation of blood) levels of substances allows the assessment of acute drug‐induced impairment and permits the measurement of medication levels for the adjustment of the drug dose or for proving drug compliance (therapeutic drug monitoring, TDM).

Xenobiotics are distributed unevenly between the liquid and cellular phase of blood [1, 2]. Consequently, a direct comparison of results derived from blood‐to‐plasma/serum concentrations, as obtained from pharmacokinetic studies, is challenging. In cases where drug concentrations approaching legal or ‘per se’ limits, or that are at the lower or upper limit of the therapeutic range, severe ramifications may ensue. Thus, the knowledge of the blood‐to‐plasma ratio (B/P; i.e., concentration of a drug in blood compared to plasma) is useful to compare drug concentrations derived from either blood or plasma. The B/P ratio of a drug is influenced by several factors, for example, volume of distribution (Vd), hematocrit value, its concentration and physiochemical properties such as lipophilicity [3, 4]. For instance, the B/P ratio of clonazepam ranges from 0.5 to 0.6, indicating that the concentrations measured in plasma are approximately twice as high as those in blood [2]. Saito et al. [5] investigated nine paired serum and blood samples from suspected intoxications with quetiapine and reported a blood‐to‐serum (B/S) ratio ranging from 0.5 to 1.1. However, the ratio increased in conjunction with elevated blood and serum quetiapine concentrations; at approximately 2500 ng/mL, the ratio reversed to >1.0. The antiepileptic drug topiramate exhibits at low therapeutic concentrations a B/P ratio which decreased from 8 to 2 as its concentration increased, suggesting a substantial and saturable binding to erythrocytes [6]. However, it should be noted that B/P ratios are usually determined in pharmacokinetic studies, where blood specimens are collected under controlled conditions and drug concentrations are mostly evaluated in plasma. In forensic laboratories, blood samples are often not characterized in detail [4].

Another consideration in TDM and forensic investigations is sample stability *in vitro*, as post‐sampling metabolism can affect drug concentrations. A change of analyte concentration can occur due to different mechanisms, for example, chemical degradation (e.g., hydrolysis), often into metabolites by reaction with enzymes or decomposition due to storage conditions (e.g., temperature) [7, 8]. Therefore, blood collection systems containing additives and preservatives, such as sodium fluoride, are used to ensure the stability of compounds. For instance, cocaine concentrations in unpreserved blood have been shown to undergo significant degradation over time. Fluoride prevents such degradation by inhibiting the process of pseudo‐cholinesterase hydrolysis of cocaine into its metabolites [9, 10]. Moreover, the addition of sodium fluoride as a preservative helps to maintain the integrity of zopiclone in collected blood samples by reducing pseudo‐cholinesterase activity [11, 12]. Two different studies examined the effect of sodium fluoride on the concentration of Δ^9^‐tetrahydrocannabinol and its metabolites in serum and plasma samples and showed that cannabinoid concentrations were decreased in sodium fluoride‐stabilized samples [13, 14]. Furthermore, Wiedfeld et al. [14] observed that mean amphetamine concentration was significantly higher in samples with the addition of fluoride. In contrast, data on the influence of sodium fluoride on serum concentrations of drugs such as antidepressants or antiepileptics remain scarce. However, in the case of bupropion, a study found that refrigeration (4°C) or freezing (−20°C) and sodium fluoride as a preservative may delay degradation of bupropion in postmortem blood [15]. Furthermore, the use of fluoride in postmortem blood reduced the rapid conversion of clonazepam, nitrazepam, and flunitrazepam to their respective 7‐amino metabolite; in some cases, however, the conversion has already occurred prior to sample collection [16]. Therefore, the use of sodium fluoride can not only inhibit ester hydrolysis, but may also reduce certain postmortem changes, for example, some microbial degradation processes, which can affect substance concentrations in postmortem material [17]. When used as a stabilizing reagent, fluoride can promote hemolysis leading to the degradation of erythrocytes [18]. The hemoglobin content of the cells is released and can be distributed into the non‐cellular fraction of blood resulting in the dilution of plasma and a discoloration of the material interfering with, for instance, spectrophotometric or immunoassay tests [18, 19].

The use of fluoride‐stabilized blood may potentially result in reported values that differ significantly from those in blood without the addition of fluoride and may lead to misinterpretation of drug testing results. Thus, this study was designed to examine the potential impact of sodium fluoride on the serum concentrations of commonly prescribed psychotropic drugs in order to improve the interpretation of drug testing results in clinical or forensic‐toxicological issues.

## 2 MATERIALS AND METHODS

### 2.1 Study design

For this clinical, open‐label study, patients undergoing psychiatric treatment were recruited at the Department for substance dependence and psychotherapy of the LVR Hospital in Bonn (Germany) in the period of June 2020 to June 2021. Patients were regularly treated with different psychotropic drugs (i.e., antidepressants, antipsychotics, antiepileptics, benzodiazepines; Table 1). Medications were stored at approximately 21°C. In addition to routine blood collection as part of the medical treatment, two further blood samples were collected. Eight of the patients agreed on a second collection of blood samples several days to weeks after the first blood collection. Data on medication, age, and sex of the participants were obtained. Volunteers were informed about the study and its procedures, duration, as well as possible risks in a medical consultation and provided their written informed consent. All patient data were pseudonymized before evaluation. The ethics committee of the Rhenish Friedrich Wilhelm University of Bonn approved the study design (approval no. 035/20). This clinical, open‐label study was in compliance with the ethical principles originating in the Declaration of Helsinki. The trial is registered with the German Clinical Trials Register as DRKS00021579. Cases were excluded from the data set if blood samples were not available. This resulted in 100 paired blood samples, which were analyzed by liquid chromatography–tandem‐mass spectrometry (LC–MS/MS).

**TABLE 1:** List of drugs, which were used in the therapeutic treatment of patients.

Antidepressants	Antiepileptics	Antipsychotics	Benzodiazepines & Z‐drugs
Bupropion	Pregabalin	Quetiapine	7‐aminoclonazepam
Hydroxybupropion	Levetiracetam	7‐hydroxyquetiapine	Clonazepam
Citalopram	Valproate		Diazepam
Norcitalopram			Nordazepam
Doxepin			Lorazepam
Nordoxepin			Zopiclone
Duloxetine			
Fluoxetine			
Norfluoxetine			
Mirtazapine			
Normirtazapine			
Paroxetine			
Sertraline			
Norsertraline			
Trimipramine			
Venlafaxine			
O‐desmethylvenlafaxine			

### 2.2 Blood collection

Blood samples were collected once intravenously (needle aspiration) using two 7.5 mL plain S‐Monovette^®^ plastic tubes (order number: 01.1601.001, Sarstedt AG & Co. KG, Nümbrecht, Germany). Silicate‐coated granulate beads are used in the S‐Monovette^®^ as a clotting activator. Approximately 50 mg of sodium fluoride was added to one of the collection devices before the blood sampling, whereas the other device was devoid of fluoride. The collected blood samples were centrifuged at 1250 × *g* for 5 min immediately after collection, and the supernatants with and without fluoride were transferred into separate 5 mL polypropylene cryotubes (Greiner Bio‐One GmbH, Germany). The degree of hemolysis was not quantified in the course of this study. However, a visual estimation of the color of the supernatant was performed. The supernatants were then brought to the laboratory of the Institute of Forensic Medicine Bonn under cooled conditions. Upon arrival, the samples were stored at −20°C until analysis (few hours to few days at most).

### 2.3 Chemicals and reference materials

Acetonitrile (≥99.9%), formic acid (98%–100%), 1‐chlorobutane (≥99.8%), *n*‐hexane (≥96.0%), ammonium acetate, and buffer solution (boric acid/potassium chloride/sodium hydroxide; pH 9) were purchased from Merck (Darmstadt, Germany). Ammonium formate (≥99.0%), methanol (≥99.9%), and buffer solution (citric acid/sodium hydroxide; pH 5) were obtained from Honeywell/Fluka™ (Seelze, Germany). Dichloromethane (≥99.8%) was purchased from VWR (Darmstadt, Germany). Ultrapure water was produced using the Arium^®^ Pro Water Purification system from Sartorius (Goettingen, Germany). The buffer solution (boric acid/sodium hydroxide; pH 11) was obtained from PanReac AppliChem (Darmstadt, Germany). All chemicals were at least of practical grade (p.A.) certified. Standard solutions and isotope‐labeled analogues used as internal standards were acquired from different companies including Sigma Aldrich^®^ (Steinheim, Germany), LGC GmbH (Luckenwalde, Wesel, Germany), Cerilliant (Round Rock, TX, USA), Santa Cruz Biotechnology (Dallas, TX, USA), Cayman Chemicals (Ann Arbor, MI, USA), Lipomed (Arlesheim, Switzerland), Toronto Research Chemicals (Toronto, Canada), Tokyo Chemical Industry Co. (Zwijndrecht, Belgium), and United States Pharmacopeial (Twinbrook, Rockville, MD, USA). A detailed summary of all analytes and internal standards, as well as their manufacturers, can be found in Table S1.

### 2.4 Sample preparation

All compounds were processed according to different protocols. An overview of the sample volumes and the extraction methods used is given in Table S2. The internal standard mix for each method contained a number of isotope‐labeled analytes. Serum samples were extracted either by liquid–liquid extraction or by protein precipitation (PP). After addition of the extraction agent or the precipitation reagent, the samples were mixed thoroughly (30–60 s) and centrifuged at 22,673 ×*g* for 6–10 min. The resulting supernatant was either evaporated under nitrogen (30–60°C) and subsequently reconstituted or injected directly into the LC system as an aliquot in an autosampler vial. For antiepileptic drugs, 200 μL of supernatant was further diluted with 1.3 mL of ultrapure water before injection into the LC system.

### 2.5 Instrumentation and sample analysis

For analysis of antidepressants (quetiapine and its metabolite were included in the antidepressant method), and pregabalin, an LC–MS/MS instrument consisting of an LC‐20A series high‐pressure liquid chromatography (HPLC) system (binary pump, degasser, column oven, and autosampler; Shimadzu, Duisburg, Germany) coupled to an API 4000 QTrap^®^ mass spectrometer (Applied Biosystems/Sciex, Darmstadt, Germany) with an electrospray Turbo V™ Ion source was used. Analytes were measured using positive electrospray ionization (ESI) and the multiple reaction monitoring (MRM) mode with two specific ion transitions per analyte.

Benzodiazepines, z‐substances, and selected antiepileptics (levetiracetam, valproate) were determined by an Agilent Series 1100 HPLC system from Agilent Technologies (Santa Clara, USA) connected to an API 4000 mass spectrometer (Applied Biosystems/Sciex, Darmstadt, Germany) with an electrospray Turbo V™ Ion source. For the determination of levetiracetam, benzodiazepines, and z‐substances, mass spectrometric detection operated in positive ESI and MRM mode with two specific ion transitions per analyte. For the determination of valproate, the mass spectrometer operated in negative ESI, and for identification, acetate adducts (valproate acetic acid *m/z* 202.9/142.7 [target], valproate sodium acetate *m/z* 224.9/142.7 [qualifier]) were used. The scheduled MRM™ mode was used for the analysis of benzodiazepines and z‐substances. The chromatographic settings (analytical column, gradient elution, mobile phases, injection volume) for analyte separation are listed in Table S3. Main mass spectrometric conditions for the determination of all compounds are listed in Table S4. Analyst^®^ software (Sciex, Darmstadt, Germany) was used for instrument control and data processing.

The paired patient samples were all analyzed once in a consecutive manner within the same analysis batch. In addition, one set of quality control (QC) samples with low and high concentrations (in relation to the respective calibration range) for each method was prepared and measured exemplarily in serum with and without sodium fluoride. Data evaluation of these samples and the paired patient samples was based on a routinely used calibration (matrix‐matched without fluoride). The determined deviation from the respective nominal concentration was nearly equivalent between the fluoride‐free and fluoride‐stabilized serum for most of the substances (0.88–1.18, ratio fluoride‐stabilized/fluoride‐free). In contrast, higher deviations from the nominal concentrations were observed for levetiracetam (−29%, low QC; −23%, high QC) and O‐desmethylvenlafaxine (+45%, low QC; +57%, high QC) in the fluoride‐stabilized serum, while deviations from the nominal concentrations in fluoride‐free serum were acceptable (levetiracetam: +4%, low QC; +1%, high QC; O‐desmethylvenlafaxine: +16%, low QC; ±0%, high QC).

### 2.6 Method validation

All methods have been established for routine measurements and were not specifically validated for the present study. The validation of the different methods was conducted in accordance with the guidelines of the Society of Toxicology and Forensic Chemistry (GTFCh) [20] in serum/plasma without sodium fluoride as a preservative. It should be noted that only the validation data of the analytes used for the statistical evaluation are described in this work. Data were processed using Valistat^®^ 2.0 software (ARVECON GmbH, Walldorf, Germany). Selectivity and specificity were demonstrated for each compound of the methods used. Data on the used analytical limits (limit of detection and limit of quantification), linearity, precision (i.e., repeatability and intermediate precision) and accuracy are presented in Table S5 for the drugs tested included in the statistical evaluation. For linear calibration, the weighting factor of 1/*x* ^2^ was used for the selected antidepressants and quetiapine (+7‐hydroxyquetiapine), whereas for levetiracetam, valproate, and clonazepam, no weighting factor was used. The regression coefficients (*R* ^2^) for all compounds were at least greater than 0.99. For determination of repeatability (within‐run precision) and intermediate precision (between‐run precision), both expressed as relative standard deviation (RSD), as well as the accuracy (calculated as bias in percent), QCs of two different levels (low and high) were measured in duplicate on eight consecutive days. The processed sample stability was evaluated for each substance in serum over a period of 10 to 25 h. Data on freeze/thaw stability and long‐term stability for the compounds were not collected as part of the respective method validation; however, they were obtained from the literature. Data on the used internal standards, matrix effects, and recovery are given in Table S6. The examined analytes demonstrated recoveries (≥50%, standard deviation [SD] ≤25%) and matrix effects (75%–125%, SD ≤25%) that were within the specified range according to the guidelines of the GTFCh [20]. For O‐desmethylvenlafaxine, the recovery and the matrix effects for the low QC were not within the specified range indicating ion suppression and insufficient extraction recovery but with acceptable SD (Table S6).

### 2.7 Statistical evaluation

For the metric parameter ‘*age*’, absolute frequencies, mean, SD, minimum, and maximum values were calculated. The nominal variable ‘*sex*’ was analyzed according to the number of characteristics ‘*male*’, ‘*female*’, and ‘*diverse*’. A minimum effective sample size of five paired samples (*n* = 5) was assumed per substance. In addition, only samples with concentrations within the respective calibration range were included in the statistical analysis. Concentration ratio of samples with and without fluoride was determined for each analyte. Measures of central tendency (mean, median, minimum, and maximum values) and the SD were calculated for every substance in all paired blood samples and their respective concentration ratios. Data were tested for normal distribution using the Shapiro–Wilk test (*p* > 0.05). The paired two‐tailed *t*‐test (*α* = 0.05) was performed to statistically compare the quantitative results of the fluoridated serum samples with the corresponding results of the non‐fluoridated serum samples. Data evaluation was carried out using SPSS Statistics 29 (IBM Corp., Armonk, NY, USA) and GraphPad Prism 5.01 (GraphPad Software, Inc., San Diego, CA, USA).

## 3 RESULTS

### 3.1 Patient collective

The study population consisted of 92 randomized patients. Of these, 69 were male and 23 were female. From 8 out of 92 patients, paired blood samples were collected twice at different times, with several days to weeks between the collections. This resulted in 100 paired blood samples with and without fluoride. The average age of the patients was 45 years (±12.5 years, SD), with the youngest participant being 19 years old and the oldest 65 years.

### 3.2 Analysis and evaluation of serum samples

**FIGURE 1:**
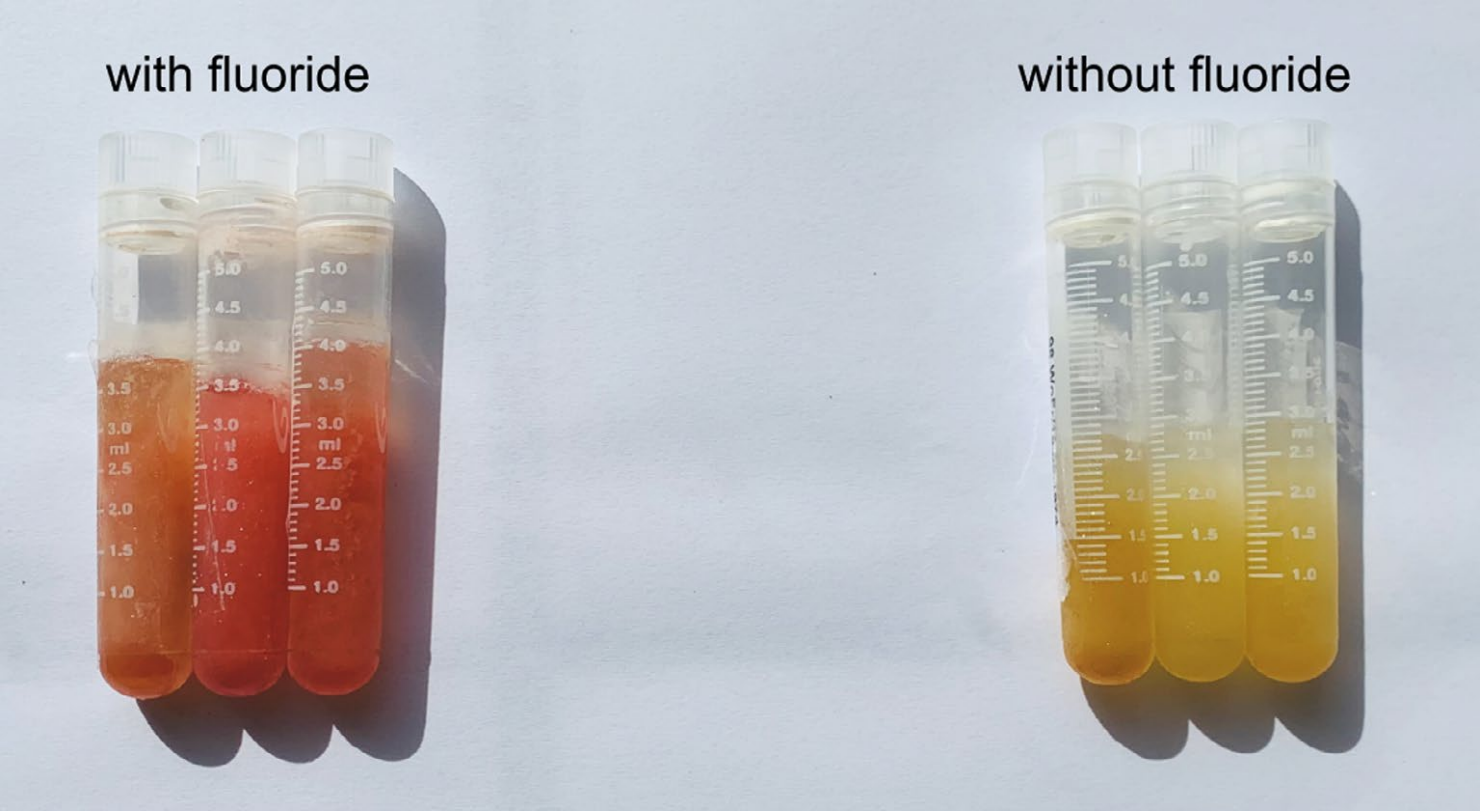
Paired serum supernatants stabilized with (left) and without sodium fluoride (right). Fluoridated serum exhibits a reddish hue in contrast to non‐fluoridated serum.

A perceivable difference was visible between the samples stabilized with fluoride and the samples without fluoride after centrifugation of the collected blood samples. Supernatants stabilized with fluoride had a reddish coloring while the non‐fluoridated supernatants showed a clear up to a yellowish color (Figure 1). The intensity of the reddish coloration varied between different blood specimens. More supernatant was obtained in the samples stabilized with fluoride than in the samples without fluoride.

Detailed descriptive data for all substances in the fluoride‐stabilized and the corresponding fluoride‐free serums (ratios and absolute concentrations) are summarized in Table 2. Not all analytes listed in Table 1 were evaluated since their sample number was too small (*n* < 5).

**TABLE 2:** Determined ratios and absolute concentrations for each compound in paired blood samples with (fluoride‐stabilized) and without (fluoride‐free) fluoride.

Compound	*n*	Ratio (fluoride‐stabilized/fluoride‐free)		*t*‐value	Fluoride‐stabilized (ng/mL)			Fluoride‐free (ng/mL)		
		Median	Range		Min	Max	Mean ± SD	Min	Max	Mean ± SD
Mirtazapine	8	0.80	0.57–0.88	−4.73*	12.3	49.8	25.8 ± 14.5	14.9	60.2	32.7 ± 16.5
Normirtazapine	8	0.77	0.53–0.88	−4.73*	5.3	27.1	12.3 ± 7.1	6.8	30.7	16.1 ± 7.9
Norsertraline	5	0.83	0.77–0.88	−1.74	12.1	101	38.2 ± 36.7	15.5	131	47.3 ± 48.2
O‐desmethylvenlafaxine	11	1.13	0.92–1.26	2.33*	29.6	386	124 ± 100	23.6	318	110 ± 83.8
Quetiapine	13	0.70	0.61–0.97	−1.97*	5.9	173	45.1 ± 48.6	8.7	177	56.5 ± 53.0
Sertraline	5	0.90	0.87–0.92	−4.40*	11.4	52.3	23.3 ± 17.1	12.6	56.4	25.7 ± 18.2
Trimipramine	5	0.83	0.75–0.85	−1.86	21.2	246	79.9 ± 93.4	25.6	305	98.9 ± 116
Venlafaxine	10	0.93	0.79–1.15	−2.86*	12.2	74.9	36.5 ± 19.6	12.9	84.0	39.5 ± 21.5
Levetiracetam	14	0.94	0.74–1.15	−1.04	1600	19,100	11,171 ± 5912	1800	20,000	11,536 ± 5722
Clonazepam	33	0.79	0.61–1.03	−11.26*	10.5	48.4	20.5 ± 8.1	14.8	53.1	25.4 ± 8.7

*Note:* The *t*‐value marked with an asterisk indicates a significant difference (*α* = 0.05).

Abbreviations: max, maximum; min, minimum; *n*, number of paired samples; SD, standard deviation.

**FIGURE 2:**
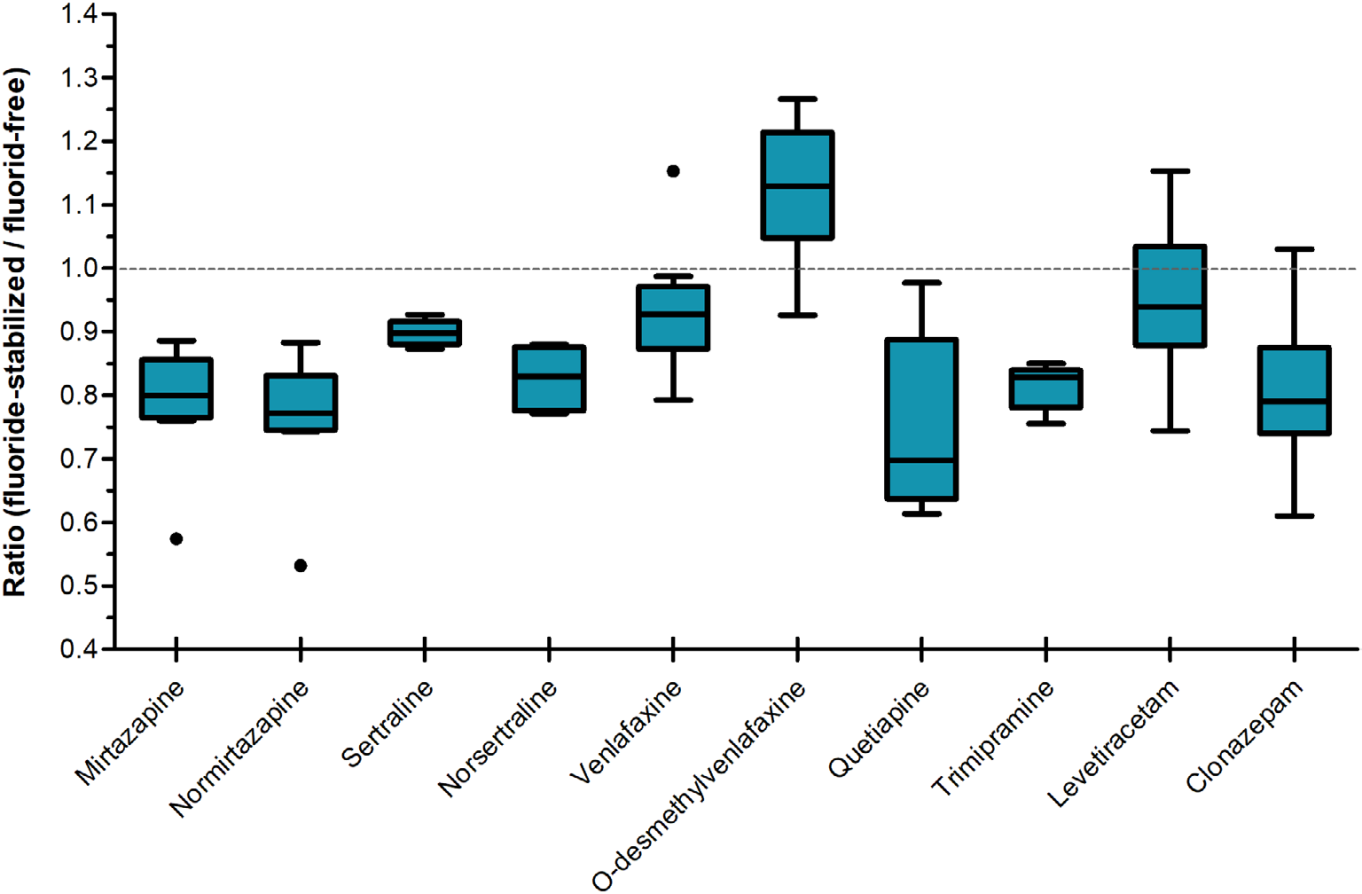
Comparison of drug concentrations expressed as the ratio between paired blood samples (fluoride‐stabilized/fluoride‐free). The dashed line represents a concentration ratio of 1.0. Ratios falling below 0.6 (mirtazapine, normirtazapine) and exceeding 1.1 (venlafaxine) are identified as outliers.

The differences between the mean analyte concentrations in the fluoride‐stabilized and the fluoride‐free samples were normally distributed, except for four compounds (norsertraline, O‐desmethylvenlafaxine, quetiapine, and trimipramine), as assessed by the Shapiro–Wilk test (Table S7). Simple outliers could be identified for mirtazapine (0.57, Figure 2), normirtazapine (0.53; Figure 2), and venlafaxine (1.15; Figure 2). The detailed results of the paired *t*‐test are presented in Table S8.

The median concentration ratio (fluoride‐stabilized/fluoride‐free) of all compounds was lowest for quetiapine (0.70, Table 2) indicating that the median concentration in the serum with fluoride was lower by 30%. In contrast, there was a 13% increase in the median concentration in the fluoride‐stabilized serum for O‐desmethylvenlafaxine compared to the fluoride‐free serum (1.13, Table 2). Only O‐desmethylvenlafaxine showed an increase in concentration in the fluoride‐stabilized sample (Figure 2). Normirtazapine, clonazepam, trimipramine, and norsertraline showed median concentration ratios between 0.77 and 0.83 (Table 2). For these four substances, the concentrations in the fluoride‐added sample were 17%–23% lower than in the non‐fluoridated sample. The median concentration ratio closest to 1 was observed for sertraline, levetiracetam, and venlafaxine (Figure 2). Their measured median concentrations in the fluoride‐stabilized serum were 6%–10% lower than in the corresponding fluoride‐free serum.

## 4 DISCUSSION

### 4.1 Comparison of drug concentrations in fluoride‐free versus fluoride‐stabilized serum

The main finding was that concentrations of most of the substances were higher in samples without fluoride than in samples stabilized with fluoride. This can be seen from the median ratios, ranging from 0.70 to 0.94 for most of the substances tested. Ideally, the mean ratio should be equal to 1, indicating no difference in concentrations between fluoride‐free and fluoride‐stabilized samples. However, when comparing substance concentrations in serum with and without the addition of fluoride as stabilizer, several aspects must be taken into account: analytical matrix effects, physicochemical properties of the compound, fluoride‐induced hemolysis, and sample and drug stability in vitro (post‐sampling metabolism).

Considering the influence of possible matrix effects, data of levetiracetam and O‐desmethylvenlafaxine must be discussed in particular. The results of the QCs for levetiracetam and O‐desmethylvenlafaxine indicate that despite adding the same drug concentration to each QC sample, lower concentrations for levetiracetam (approximately −30%) and higher concentrations for O‐desmethylvenlafaxine (approximately +60%), respectively, were observed in serum with fluoride (as described in Section 2.5). As the patient samples were always evaluated using the routinely used matrix‐matched calibration without fluoride, these observations suggest that different matrix effects in fluoride‐stabilized serum may have influenced the concentrations of both drugs in the QC as well as in the patient samples with fluoride resulting in under‐ and overestimation of the results. Consequently, the median concentration ratios of levetiracetam and O‐desmethylvenlafaxine determined for the patient samples must be considered with caution. Given the influence of matrix effects on the results for levetiracetam and O‐desmethylvenlafaxine in the patient samples, significantly lower levetiracetam concentrations and higher O‐desmethylvenlafaxine concentrations in the fluoride‐stabilized serum would have been expected, assuming that the same drug concentration is present in both matrices after the ingestion of the (parent) drug. Median concentration ratios for levetiracetam and O‐desmethylvenlafaxine were 0.94 (0.74–1.15, Table 2) and 1.13 (0.92–1.26, Table 2), respectively. In consideration of the results of the QC samples, these observations may indicate minimal deviations between the obtained patient serum with and without fluoride.

Assuming that hemolyzed serum is comparable to blood due to the leakage of red blood cell contents into serum/plasma [18], a comparison of the results obtained with the B/P or B/S ratio of the examined compounds may provide information about their distribution in the two matrices. Furthermore, the individual physiochemical properties and the Vd of the drugs examined in this study should be considered.

For levetiracetam (QC containing fluoride: approximately −30%), concentration ratios in patient samples imply a slightly higher concentration in fluoride‐stabilized samples. Levetiracetam is not extensively metabolized in humans [21]. The metabolic pathway is the enzymatic hydrolysis of its acetamide group into its pharmacologically inactive metabolite UCB L057 [21]. In fact, the hydrolysis is mediated by serine‐type esterases in various tissues, including erythrocytes [21]. Serine‐type esterases are a broad class of enzymes having the ability to hydrolyze ester or peptide bonds (e.g., amides) [22]. Two different studies investigated the effect of different esterase inhibitors on the hydrolysis of levetiracetam including sodium fluoride in human whole blood [23, 24]. No relevant inhibition of the enzymatic hydrolysis was exhibited by the use of fluoride (UCB L057 formed 91.4 ± 4.1 pmol/min/mL blood) compared to the use of the control sample (1% water; UCB L057 formed 84.4 ± 12.8 pmol/min/mL blood) [23]. The authors suggested from the results that levetiracetam is not a suitable substrate for pseudo‐cholinesterase [23]. Considering its B/P ratio ranging from 0.8 to 0.9 [2], levetiracetam levels are slightly lower in blood than in plasma, indicating that it does not strongly bind to erythrocytes and is primarily distributed in plasma. Given its low binding affinity for plasma proteins (3.4%) and a Vd ranging from 0.5 to 0.7 L/kg, which is close to the volume of total body water, levetiracetam is well distributed in aqueous compartments of the body without significant sequestration in blood cells or tissues [23, 25].

The median concentration ratio of 1.13 in patient samples obtained for O‐desmethylvenlafaxine may suggest the potential for higher levels in fluoridated serum. With regard to the results of the QCs, however, different matrix effects in serum with fluoride may have affected the results obtained. In consideration of matrix‐induced overestimation (approximately +60%), the concentration ratio observed for the patient samples (1.13, Table 2) implies a slightly lower concentration in fluoride‐stabilized samples. The Vd for venlafaxine (7.5 ± 3.7 L/kg) and for O‐desmethylvenlafaxine (5.7 ± 1.8 L/kg) and the low plasma protein binding of approximately 27% facilitate their diffusion across cell membranes, resulting in their distribution not only in plasma but also into erythrocytes or body tissues having a higher lipid content than plasma [26]. To the best of the authors' knowledge, there is no available data on the B/P ratio of O‐desmethylvenlafaxine to determine its distribution between blood and plasma. However, the reported B/P ratio of 1.17 for its parent compound venlafaxine indicates a higher concentration in blood compared to plasma [27]. This contradicts the data collected here for venlafaxine, which did not show a median concentration ratio greater than 1 in the fluoride‐stabilized material (0.93, Table 2), provided that serum with fluoride is comparable to blood rather than to plasma due to hemolysis. However, it should be noted that a single outlier was observed, exhibiting a median concentration ratio of 1.1 (Figure 2), which is consistent with the mentioned B/P of 1.17.

In contrast to levetiracetam and O‐desmethylvenlafaxine, the results of the QC samples for mirtazapine, sertraline (and their respective metabolites), quetiapine, clonazepam, and trimipramine exhibited no considerable deviation in drug concentrations between fluoride‐free and fluoride‐stabilized serum, indicating that analytical influences should rather be negligible. Therefore, the obtained results on the paired patient samples indicated that physicochemical properties and distribution of the analytes as well as hemolysis in fluoride‐stabilized serum may have influenced the concentrations in both matrices. The B/P ratios for trimipramine (0.8 [2]), clonazepam (0.5 to 0.6 [2]), and the B/S ratio for quetiapine (0.74 [28]) indicate a consistent partitioning into serum/plasma and a low association with erythrocytes, implying higher drug concentrations in the non‐cellular blood fraction. On the other hand, the literature data [2] for mirtazapine (1.0, B/P) and sertraline (1.1–1.2, B/P) differ marginally from the median concentrations determined in this study (0.8, mirtazapine; 0.9, sertraline, Table 2). Considering their B/P ratios, it would have been expected that mirtazapine and sertraline (and their respective metabolites) concentrations would be higher or at least equivalent in serum with fluoride compared to serum without fluoride, provided that hemolyzed serum is comparable to blood. Antidepressants are generally considered to be amphiphilic molecules, with the capacity to dissolve in the aqueous phase, though they are equally soluble in the lipid part of cell membranes [29]. Their ability to permeate membranes allows for their presence in plasma as well as in cell membranes and cytoplasm, where they reach a state of equilibrium [29]. Despite sertraline's high plasma protein binding affinity (98%–99% [30, 31]), it accumulates in the inner monolayer of the erythrocyte plasma membrane [32]. Furthermore, its lipophilicity enables its partitioning into erythrocytes, thereby increasing its concentration in blood relative to plasma which is consistent with its B/P ratio exceeding 1. In summary, the results obtained in this study are, to a limited extent, consistent with the literature data on B/P or B/S ratios, under the assumption that hemolyzed serum is comparable to blood. Thus, the results can provide guidance for the interpretation of drug concentrations in hemolyzed serum/plasma. Nevertheless, caution is still required due to the severe matrix effects observed for levetiracetam and O‐desmethylvenlafaxine in fluoride‐stabilized QC samples.

Sodium fluoride (e.g., used as a stabilizer) induces hemolysis, which, in addition to the already mentioned matrix effects, impacts the interpretation of the results. The effects of hemolysis can be associated with multiple effects. Firstly, concentrations of drugs present at higher concentrations in plasma than in erythrocytes can be falsely decreased. Secondly, concentrations of substances in hemolyzed serum/plasma that are normally present in higher concentrations within erythrocytes can be falsely elevated. Both can be attributed to the caused shift in erythrocyte components and dilution of the serum/plasma due to hemolysis [33, 34]. In fact, the fluoridated serum samples in this study yielded more supernatant compared to the non‐fluoridated samples, indicating an increase in the serum volume. Additionally, the fluoridated samples exhibited varying intensities of red discoloration and different degrees of hemolysis (moderate to severe). These observations can be posited to the degradation of the erythrocytes and the subsequent release of the cell content into the plasma. Considering that fluoride‐induced hemolysis caused a dilution of the serum, it seems reasonable that the distribution of the compounds between the cellular and fluid phase of blood changed, thereby influencing their concentration.

To prevent drug degradation in vitro, collected blood samples were immediately centrifuged and supernatants were stored at −20°C prior to analysis. In general, most of the investigated drugs were reported to be stable under different storage conditions, for example, at −20°C for days to 1 year or several freeze/thaw cycles in serum or plasma [35]. Even at room temperature, drugs such as quetiapine, sertraline, venlafaxine, and their respective metabolites were stable in spiked plasma samples over a period of 7 days [36]. However, to the best of the authors' knowledge, there is no available data on in vitro stability of psychotropic drugs for TDM in fluoride‐stabilized serum. Moreover, the herein used study design does not allow to conclude whether the investigated substances are stable in the presence or absence of sodium fluoride in serum. The function of fluoride as a preservative, specifically as an inhibitor of pseudo‐cholinesterases, suggests that substances containing ester bonds are stabilized by the addition of fluoride (e.g., cocaine) [9]. Given the absence of ester bonds in the chemical structure of the compounds evaluated in this study, it can be assumed that the presence or absence of fluoride does not contribute to the chemical stability of the substances in the collected blood sample.

### 4.2 Limitations of the study

The herein presented study exhibited several limitations that may affect the interpretation of the results. First of all, the lack of method validation for the determination of analytes in serum with fluoride represents a substantial limitation of this study. This includes linearity, testing for stability, and matrix effects. The evaluation of the results obtained in serum with fluoride was not conducted through the implementation of a matrix‐matched calibration. Rather, it was performed by using calibration levels in serum/plasma devoid of fluoride (as described in Section 2.6). Calibration is often performed on one available blank matrix sample, and all other matrix samples are calculated from this calibration curve in order to save time and resources. For instance, Montenarh et al. [37] demonstrated comparable results of the QC levels for trimipramine in whole blood, plasma, and serum using one plasma calibration curve. Moreover, the quantitative determination of an analysis batch was always verified using matrix‐matched QC levels (in serum without fluoride) for each method. No valid conclusions can be made regarding the chemical stability of the drugs examined in fluoride‐stabilized serum, as the stability tests (e.g., freeze/thaw or long‐term stability) during method validation were also only carried out for substances in serum/plasma without fluoride, or data were taken from the literature, which examined blood, plasma, or serum without the specific addition of fluoride. The validated matrix effects and the corresponding SD for most of the compounds examined in serum/plasma without fluoride were within the acceptable range, indicating minimal impact of the sample matrix on the ionization and detection of the target analyte (Table S6). However, the QC results for levetiracetam and O‐desmethylvenlafaxine assume matrix effects due to the fluoride‐induced hemolysis that might not be compensated by the use of an isotopically labeled internal standard. Therefore, testing for matrix effects (e.g., according to the method of Matuszewski et al. [38]) in fluoridated serum would be required for a definitive statement. In general, not all possible pre‐analytical variations that may arise in a blood sample, such as those resulting from interfering factors like lipemia, hemolysis, or icterus, can be fully represented in a method validation, although they may have an influence on analyte detection and recovery. This limitation stems from several factors: the inherent complexity and variability of pre‐analytical variations, and the practical constraints associated with the validation process, which is often resource‐intensive. Moreover, validation guidelines generally mandate representative validation but do not necessitate the testing of every conceivable pre‐analytical variation. Consequently, laboratories prioritize on validating methods under standard conditions.

Fluoride‐induced hemolysis is considered to be one of the major aspects affecting drug concentrations; however, neither the degree of hemolysis or the hematocrit value was determined using quantitative methods, as both are not usually measured in forensic routine casework.

For certain substances, such as sertraline, the number of paired samples was limited (*n* = 5). Moreover, the exemplary measurements of one set of paired QC levels for determination of different matrix effects of serum with and without sodium fluoride appear to be insufficient for deriving final conclusions. Consequently, further investigations using larger sample sets are recommended to ensure the reproducibility of the results.

In a clinical context, the use of the same sample material for the analysis of various compounds is unlikely as standardized procedures are used (e.g., ethylenediaminetetraacetic acid tubes for analysis of immunosuppressive drugs). In contrast, this is more challenging in forensic scenarios. For instance, the rejection of hemolytic blood samples obtained during police investigations for forensic toxicology testing is not a viable option, as it can occur for some routine clinical chemistry tests. Usually, the blood sample is collected close to the time of an incident (e.g., in case of driving under the influence of drugs), which means that acute drug effects can still be inferred. Additionally, specimens sent by mail from investigating authorities to toxicological laboratories often endure several days, under nonspecific provisions, which may affect sample and drug stability. Furthermore, there is a wide range of compounds (from illicit to licit drugs) which are tested using the same sample material obtained from a specific blood collection system, with no specific distinction between the analytes which may lead to different interpretations in the toxicological assessment of drug‐induced impairment.

## 5 CONCLUSIONS

In this investigation, it was observed that the concentrations of most of the substances in paired serum samples of psychiatric patients were lower in fluoride‐stabilized serum in contrast to serum without fluoride addition. This observation may result inter alia from fluoride‐induced hemolysis and analytical influences affecting the drug concentration in serum with fluoride. Caution should be exercised when considering the mean concentration ratios of patient samples for levetiracetam and O‐desmethylvenlafaxine based on the results of the paired set of QC samples, which indicate strong matrix effects in serum due to the addition of fluoride. In cases were fluoride‐free serum is not available, and fluoride‐stabilized serum is used for drug analysis, factors such as the B/P or B/S ratio for the examined drug should be considered to account for any differences in the distribution of the drug (or its metabolite) in blood constituents. Consequently, the influence of stabilizers added to blood samples should be kept in mind when using common routine applications for a precise interpretation of drug results.

## CONFLICT OF INTEREST STATEMENT

The authors declare no conflicts of interest.

## Supporting information


Appendix S1.

## Data Availability

The data underlying this article are available in the article and in its online supplementary material. More detailed data on the patient chohort cannot be shared publicly due to the privacy of the individuals who participated in the study.
