# Supplementary material for: Effects of sodium fluoride on serum concentrations of selected psychotropic drugs
Source: J Forensic Sci. 2025 May 25;70(5):1939–48. doi: 10.1111/1556-4029.70081 (PMC12424277; doi:10.1111/1556-4029.70081)
Supplement: Supplementary file 1 — Appendix S1. [file JFO-70-1939-s001.docx]

# **Supplemental Information**

Table S1 Table for all compounds, internal standards and their manufacturers.

| **Method** | **Compound** | **Manufacturer** |
| --- | --- | --- |
| Antiepileptics | Carbamazepine | LGC (Luckenwalde, Germany) |
|  | 10,11-dihydro-10-hydroxycarbamazepine | LGC (Luckenwalde, Germany) |
|  | Felbamate | Sigma Aldrich (St. Louis, MO, USA) |
|  | Lamotrigine | LGC (Luckenwalde, Germany) |
|  | Levetiracetam | Sigma Aldrich (St. Louis, MO, USA) |
|  | Oxcarbazepine | LGC (Luckenwalde, Germany) |
|  | Carbamazepine-d10 | Sigma Aldrich (St. Louis, MO, USA) |
|  | Levetiracetam-d6 | Sigma Aldrich (St. Louis, MO, USA) |
|  | Gabapentin | Sigma Aldrich (St. Louis, MO, USA) |
|  | Gabapentin-d10 | Cerilliant (Round Rock, TX, USA) |
|  | Pregabalin | Sigma- Aldrich (St. Louis, MO, USA) |
|  | Pregabalin-d6 | Cerilliant (Round Rock, TX, USA) |
|  | Ritalinic acid | Sigma Aldrich (St. Louis, MO, USA) |
|  | (±) threo-Ritalinic acid-d10 HCl | Cerilliant (Round Rock, TX, USA) |
|  | Topiramate | LGC (Luckenwalde, Germany) |
|  | Topiramate-d12 | Cerilliant (Round Rock, TX, USA) |
|  | Vigabatrin | Cerilliant (Round Rock, TX, USA) |
|  | Valproate | LGC (Luckenwalde, Germany) |
|  | Valproate-d6 | Cerilliant (Round Rock, TX, USA) |
| Benzodiazpines and Z-Drugs | Hydroxyethylflurazepam | LGC (Luckenwalde, Germany) |
|  | 2-hydroxyethylflurazepam-d4 | LGC (Luckenwalde, Germany) |
|  | 3-hydroxybromazepam | LGC (Luckenwalde, Germany) |
|  | 7-aminoclonazepam | LGC (Luckenwalde, Germany) |
|  | 7-aminoflunitrazepam | LGC (Luckenwalde, Germany) |
|  | 7-aminoflunitrazepam-d7 | LGC (Luckenwalde, Germany) |
|  | α-hydroxyalprazolam | LGC (Luckenwalde, Germany) |
|  | α-hydroxymidazolam | LGC (Luckenwalde, Germany) |
|  | Alprazolam | LGC (Luckenwalde, Germany) |
|  | Alprazolam-d5 | LGC (Luckenwalde, Germany) |
|  | Bromazepam | LGC (Luckenwalde, Germany) |
|  | Clobazam | LGC (Luckenwalde, Germany) |
|  | Clonazepam | LGC (Luckenwalde, Germany) |
|  | Delorazepam | LGC (Luckenwalde, Germany) |
|  | Diazepam | LGC (Luckenwalde, Germany) |
|  | Diazepam-d5 | LGC (Luckenwalde, Germany) |
|  | Diclazepam | LGC (Luckenwalde, Germany) |
|  | Etizolam | LGC (Luckenwalde, Germany) |
|  | Etizolam-d3 | LGC (Luckenwalde, Germany) |
|  | Flunitrazepam | LGC (Luckenwalde, Germany) |
|  | Flunitrazepam-d7 | LGC (Luckenwalde, Germany) |
|  | Flurazepam | LGC (Luckenwalde, Germany) |
|  | Lorazepam | LGC (Luckenwalde, Germany) |
|  | Lormetazepam | LGC (Luckenwalde, Germany) |
|  | Medazepam | Cerilliant (Round Rock, TX, USA) |
|  | Methylclonazepam | LGC (Luckenwalde, Germany) |
|  | Midazolam | LGC (Luckenwalde, Germany) |
|  | N-desalkylflurazepam | LGC (Luckenwalde, Germany) |
|  | N-desmethylclobazam | LGC (Luckenwalde, Germany) |
|  | Nitrazepam | LGC (Luckenwalde, Germany) |
|  | Nordiazepam (Desmethyldiazepam) | LGC (Luckenwalde, Germany) |
|  | Norflunitrazepam (Desmethylflunitrazepam) | LGC (Luckenwalde, Germany) |
|  | Oxazepam | LGC (Luckenwalde, Germany) |
|  | Pyrazolam | LGC (Luckenwalde, Germany) |
|  | Temazepam | LGC (Luckenwalde, Germany) |
|  | Temazepam-d5 | LGC (Luckenwalde, Germany) |
|  | Tetrazepam | LGC (Luckenwalde, Germany) |
|  | Triazolam | LGC (Luckenwalde, Germany) |
|  | Zaleplon | LGC (Luckenwalde, Germany) |
|  | Zolpidem | LGC (Luckenwalde, Germany) |
|  | Zopiclone | LGC (Luckenwalde, Germany) |
| Antidepressants | Agomelatine | Sigma Aldrich (St. Louis, MO, USA) |
|  | Amitriptyline | Sigma Aldrich (Munich, Germany) |
|  | Amitriptyline-N-Oxid | TCI (Zwijndrecht, Belgium) |
|  | Bupropion | Cerilliant (Round Rock, TX, USA) |
|  | Bupropion-d9 | Sigma Aldrich (Steinheim, Germany) |
|  | Citalopram | Sigma Aldrich (Steinheim, Germany) |
|  | Citalopram-d6 | Sigma Aldrich (Steinheim, Germany) |
|  | Clomipramine | Sigma Aldrich (Steinheim, Germany) |
|  | Desipramine | Sigma Aldrich (Steinheim, Germany) |
|  | Dosulepin (Dothiepin) | Sigma Aldrich (Steinheim, Germany) |
|  | Doxepin | Sigma Aldrich (Steinheim, Germany) |
|  | Doxepin-d3 | Sigma Aldrich (Steinheim, Germany) |
|  | Duloxetine | Sigma Aldrich (Steinheim, Germany) |
|  | Fluoxetine | Santa Cruz Biotechnology (Heidelberg, Germany) |
|  | Fluvoxamine | Sigma Aldrich (Steinheim, Germany) |
|  | Hydroxybupropion | Sigma Aldrich (St. Louis, MO, USA) |
|  | Imipramine | Santa Cruz Biotechnology (Heidelberg, Germany) |
|  | Maprotiline | Sigma Aldrich (Steinheim, Germany) |
|  | Mianserin | Cerilliant (Round Rock, TX, USA) |
|  | Mirtazapine | LGC Standards (Wesel, Germany) |
|  | Moclobemide | Sigma Aldrich (Steinheim, Germany) |
|  | N-desmethylcitalopram | Sigma Aldrich (Steinheim, Germany) |
|  | N-desmethylclomipramine | Sigma Aldrich (Steinheim, Germany) |
|  | N-desmethylmirtazapin | Sigma Aldrich (Steinheim, Germany) |
|  | N-desmethyltrimipramine | Sigma Aldrich (Steinheim, Germany) |
|  | Nordoxepin | Sigma Aldrich (Steinheim, Germany) |
|  | Norfluoxetin Oxalat | Sigma Aldrich (Munich, Germany) |
|  | Normianserin | Sigma Aldrich (Steinheim, Germany) |
|  | Desmethylsertralinee | Sigma RBI (Massachusetts, MA, USA) |
|  | Nortriptyline | Sigma Aldrich (Steinheim, Germany) |
|  | O-desmethylvenlafaxine | Sigma Aldrich (Steinheim, Germany) |
|  | Opipramol | Sigma Aldrich (Steinheim, Germany) |
|  | Paroxetine | Toronto Research Chemicals Inc. (Toronto, Canada) |
|  | Quetiapine | Sigma Aldrich (Steinheim, Germany) |
|  | Reboxetine | LGC Standards (Wesel, Germany) |
|  | Sertraline | Sigma Aldrich (Steinheim, Germany) |
|  | Sertraline-d3 | Sigma Aldrich (Steinheim, Germany) |
|  | Tranylcypromine | Sigma Aldrich (Steinheim, Germany) |
|  | Trazodone | Sigma Aldrich (St. Louis, MO, USA) |
|  | Trimipramine | Sigma Aldrich (Steinheim, Germany) |
|  | Trimipramine-d3 | Sigma Aldrich (Steinheim, Germany) |
|  | Venlafaxine | Sigma Aldrich (Steinheim, Germany) |
|  | 7-hydroxyquetiapine | Sigma Aldrich (St. Louis, MO, USA) |

Table S2 Overview of the used sample preparation methods.

| **Substance class** | **Compound** | Sample volume [µL] | Extraction method | Solvent used for extraction |
| --- | --- | --- | --- | --- |
| Antidepressants (+Quetiapine) |  | 200 | LLE | *n*-Hexane/dichloromethane (4:1) |
| Antiepileptics | Pregabalin | 100 | PP | 2 mM ammonium acetate in methanol |
|  | Levetiracetam | 10 | PP | Dichloromethane |
|  | Valproate | 100 | PP | 10 mM ammonium acetate in  deionized water/methanol (3:97); 0.1%  acetic acid |
| Benzodiazepines &  Z-Drugs |  | 200 | LLE | 1-chlorobutane |

LLE = Liquid-liquid extraction

PP = Protein precipitation

Table S3 Chromatographic settings of the analytical methods used.

| **Method** | **Compound** | **Analytical column** | **Injection volume [µL]** | **Mobile phase** |
| --- | --- | --- | --- | --- |
| Antidepressants  (+Quetiapine) |  | Allure^®^ PFP Propyl 5 μm 60Å (50 x 2.1 mm), Restek | 10 | A: 1 M ammonium formate, formic acid, aqua dem., 2:2:996 (v/v/v)  B: 1 M ammonium formate, formic acid, acetonitrile, 2:2:996 (v/v/v) |
|  |  |  |  |  |
|  |  |  |  |  |
|  |  |  |  |  |
| Antiepileptics | Pregabalin | Gemini^®^ 3 μm NX-C18 110Å (150 x 2 mm), Phenomenex | 5 | A: 1 M formic acid, aqua dem., 2:998 (v/v)  B: 1 M formic acid, methanol, 2:998 (v/v) |
|  |  |  |  |  |
|  |  |  |  |  |
|  |  |  |  |  |
|  |  |  |  |  |
|  | Levetiracetam | Luna^®^ 5 µm C8 100Å (150 x 3.0 mm), Phenomenex | 5 | A: 1 M ammonium formate, formic acid, aqua dem., 2:2:996 (v/v/v)  B: 1 M ammonium formate, formic acid, acetonitrile, 2:2:996 (v/v/v) |
|  |  |  |  |  |
|  |  |  |  |  |
|  |  |  |  |  |
|  |  |  |  |  |
|  |  |  |  |  |
|  | Valproate | ZORBAX^®^ Eclipse 5 μm XDB-C18, 80Å (150 x 4.6 mm), Agilent | 10 | A: 10 mM ammonium acetate, 0.1% acetic acid, aqua dem./methanol 95:5 (v/v)  B: 10 mM ammonium acetate, 0.1% acetic acid, aqua dem../methanol 3:97 (v/v) |
|  |  |  |  |  |
|  |  |  |  |  |
|  |  |  |  |  |
|  |  |  |  |  |
|  |  |  |  |  |
| Benzodiazepines  & Z-Drugs |  | Synergi^®^ 4 μm MAX-RP C18, 80Å (150 x 2 mm), Phenomenex | 10 | A: 5 mM ammonium formate, 0.02% formic acid, aqua dem./methanol 90:10 (v/v)  B: 5 mM ammonium formate, methanol |
|  |  |  |  |  |
|  |  |  |  |  |
|  |  |  |  |  |
|  |  |  |  |  |
|  |  |  |  |  |
|  |  |  |  |  |
|  |  |  |  |  |
|  |  |  |  |  |
|  |  |  |  |  |

Table S4 Mass spectrometric instrument parameters.

| **Parameter** | **Method** | | | | |
| --- | --- | --- | --- | --- | --- |
|  |  | Antiepileptics | | |  |
|  | Antidepressants (+Quetiapine) | Pregabalin | Levetiracetam | Valproate | Benzodiazepines & Z-Drugs |
| Collision gas | Nitrogen | Nitrogen | Nitrogen | Nitrogen | Nitrogen |
| Curtain gas | 10 psi | 20 psi | 30 psi | 30 psi | 25 psi |
| Nebulizer Gas (GS 1) | 40 psi | 40 psi | 40 psi | 40 psi | 60 psi |
| Heater Gas (GS 2) | 60 psi | 60 psi | 60 psi | 60 psi | 50 psi |
| Ion spray voltage | 4500 V | -4500 V | 5500 V | 5500 V | 4500 V |
| Ion spray temperature | 425°C | 400°C | 550°C | 550°C | 490°C |

Table S5 Data on limit of detection (LoD), limit of quantification (LoQ), linearity, precision and accuracy of the drugs tested included for evaluation.

|  | LoD  [ng/mL] | LoQ  [ng/mL] | Calibration range [ng/mL] | Repeatability  (as RSD*) [%] | | Intermediate precision  (as RSD*) [%] | | Accuracy  (as Bias) [%] | |
| --- | --- | --- | --- | --- | --- | --- | --- | --- | --- |
|  | | | | Low QC^†^ | High QC^†^ | Low QC^†^ | High QC^†^ | Low QC^†^ | High QC^†^ |
| Mirtazapine | 0.5 | 1.4 | 5 – 750 | 8.2 | 8.9 | 9.4 | 9.9 | -5.7 | -5.2 |
| Normirtazapine | 0.3 | 0.9 | 1 – 750 | 13 | 5.8 | 13 | 11 | -4.5 | +1.6 |
| Norsertraline | 0.5 | 0.5 | 1 – 750 | 12 | 2.7 | 15 | 4.0 | +0.6 | -2.2 |
| O-desmethylvenlafaxine | 1.6 | 4.9 | 5 – 750 | 17^‡^ | 18^‡^ | 23^‡^ | 18^‡^ | -11 | +12 |
| Quetiapine | 0.4 | 1.9 | 5 – 750 | 12 | 15 | 16^‡^ | 19^‡^ | -0.6 | +6.1 |
| Sertraline | 0.5 | 1.7 | 5 – 750 | 8.9 | 6.3 | 8.9 | 6.3 | -1.4 | +1.8 |
| Trimipramine | 0.3 | 1.0 | 1 – 750 | 3.2 | 4.5 | 4.9 | 6.8 | -0.9 | +3.3 |
| Venlafaxine | 0.9 | 2.8 | 5 – 750 | 13 | 5.8 | 13 | 11 | -4.5 | +1.6 |
| Levetiracetam | 9.4 | 11.7 | 1,000 – 20,000 | 2.8 | 1.5 | 2.8 | 2.0 | +1.1 | -2.1 |
| Clonazepam | 2.5 | 8.9 | 10 – 1,000 | 8.5 | 8.7 | 11 | 10 | -3.2 | -6.8 |

*RSD =relative standard deviation

^†^QC = quality control

^‡^ = not in accordance with acceptance criteria (RSD ≤15%, 20% near LoQ)

Table S6 Data on internal standards, matrix effects and recoveries of the drugs tested included for evaluation.

| Compound | Internal standards | Matrix effect (±SD) [%] | | Recovery (±SD) [%] | |
| --- | --- | --- | --- | --- | --- |
|  | | Low QC* | High QC* | Low QC* | High QC* |
| Mirtazapine | Doxepin-d3 | 124 ± 12 | 94 ± 7.1 | 89 ± 9.8 | 91 ± 5.2 |
| Normirtazapine | Doxepin-d3 | 95 ± 12 | 92 ± 5.1 | 83 ± 6.9 | 95 ± 9.1 |
| Norsertraline | Sertraline-d3 | 89 ± 11 | 100 ± 16 | 97 ± 17 | 93 ± 5.3 |
| O-desmethylvenlafaxine | Citalopram-d6 | 37^†^ ± 2.8 | 99 ± 6.9 | 29^‡^ ± 4.8 | 37^‡^ ± 2.8 |
| Quetiapine | Citalopram-d6 | 88 ± 7.5 | 99 ± 8.4 | 95 ± 14 | 95 ± 3.7 |
| Sertraline | Sertraline-d3 | 94 ± 19 | 98 ± 10 | 94 ± 25 | 96 ± 7.4 |
| Trimipramine | Trimipramine-d3 | 107 ± 14 | 92 ± 8.1 | 95 ± 15 | 100 ± 8.3 |
| Venlafaxine | Doxepin-d3 | 103 ± 12 | 100 ± 8.7 | 89 ± 10. | 90 ± 10 |
| Levetiracetam | Levetiracetam-d6 | 123 ± 3.6 | 108 ± 2.8 | 52 ± 2.6 | 54 ± 3.6 |
| Clonazepam | Flunitrazepam-d7 | 117 ± 25 | 100 ± 10 | 80 ± 8.6 | 92 ± 21 |

^*^QC = quality control

^† =^ not in accordance with acceptance criteria (75 – 125%, SD ≤25%)

^‡^ = not in accordance with acceptance criteria (≥ 50%, SD ≤25%)

Table S7 Results of the Shapiro-Wilk test.

|  | Shapiro-Wilk | | | |
| --- | --- | --- | --- | --- |
| Compound | **n** | **Statistic** | **df** | **Significance** |
| Mirtazapine | 8 | 0.945 | 8 | 0.657 |
| Normirtazapine | 8 | 0.893 | 8 | 0.250 |
| Norsertraline | 5 | 0.657 | 5 | **0.003*** |
| O-desmethylvenlafaxine | 11 | 0.747 | 11 | **0.002*** |
| Quetiapine | 13 | 0.850 | 13 | **0.028*** |
| Sertraline | 5 | 0.925 | 5 | 0.566 |
| Trimipramine | 5 | 0.700 | 5 | **0.010*** |
| Venlafaxine | 10 | 0.967 | 10 | 0.863 |
| Levetiracetam | 14 | 0.937 | 14 | 0.377 |
| Clonazepam | 33 | 0.984 | 33 | 0.884 |

**p* < 0.05

df = degrees of freedom.

Table S8 Main results of the paired Student’s t-test per compound. Calculated bold t-values with an asterisk (*) indicate significant difference tested on a significance level of α = 0.05 (two-sided) using a paired Student’s t-test.

|  | Paired Student’s *t*-test | | | |
| --- | --- | --- | --- | --- |
| Compound | **n** | **mean_Diff_** | **SD_Diff_** | ***t*-value** |
| Mirtazapine | 8 | -6.85 | 4.08 | **-4.73*** |
| Normirtazapine | 8 | -3.73 | 2.22 | **-4.73*** |
| Sertraline | 5 | -2.42 | 1.22 | **-4.40*** |
| Norsertraline | 5 | -9.16 | 11.7 | -1.74 |
| Trimipramine | 5 | -18.9 | 22.6 | -1.86 |
| Quetiapine | 13 | -11.3 | 11.0 | **-1.97*** |
| Venlafaxine | 10 | -3.06 | 3.37 | **-2.86*** |
| O-desmethylvenlafaxine | 11 | 13.8 | 19.7 | **2.33*** |
| Levetiracetam | 14 | -0.36 | 1.30 | -1.04 |
| Clonazepam | 33 | -4.82 | 2.45 | **-11.26*** |

n = number of paired samples

SD = standard deviation

Diff = difference
